# Evaluation of awareness about primary immunodeficiencies among physicians before and after implementation of the educational program: A longitudinal study

**DOI:** 10.1371/journal.pone.0233342

**Published:** 2020-05-29

**Authors:** Tetyana Hariyan, Maria Kinash, Roman Kovalenko, Oksana Boyarchuk

**Affiliations:** 1 Department of Children’s Diseases and Pediatric Surgery, I.Horbachevsky Ternopil National Medical University, Ternopil, Ukraine; 2 Faculty of Social Sciences and Social Technologies, National University of Kyiv-Mohyla Academy, Kyiv, Ukraine; Nazareth Hospital, UNITED STATES

## Abstract

Increasing physicians’ awareness is one of the main ways to improve early diagnosis of rare diseases. A survey among physicians of different specialties to evaluate the knowledge about primary immunodeficiencies (PID) was conducted in 2016 and in 2019 –before and after the implementation of an educational program. We compare responses from 82 doctors who participated in the 2016 survey, and 67 doctors who have taken part in the survey in 2019: pediatricians, general practitioners / family physicians and physicians of pediatric sub specialties. The percentage of correct answers to all survey questions after the implementation of the educational program has significantly increased (79.0% in 2019 versus 58.3% in 2016, P<0.0001). This increase in the percentage of correct answers was noted among the surveyed doctors of all specialties. Particular progress was found among pediatricians, who have achieved more than 80% of correct answers. In 2019 the doctors demonstrated better knowledge on the warning signs of PID and specific features of Nijmegen breakage syndrome, DiGeorge syndrome and ataxia-telangiectasia syndrome. Thus, the implementation of an educational program improved physicians’ awareness of PIDs, and will contribute to early detection of PIDs and their medical care.

## Introduction

The necessity of early diagnosis of rare diseases is well recognized worldwide. Rapid development of genetic research leads to discovery of new diseases previously unknown to general practitioners. Therefore, educating the doctors is one of the main ways to increase awareness of these diseases and to improve their early diagnosis.

Primary immunodeficiencies (PIDs), which today comprise more than 430 diseases, are rare diseases, even though with their number increasing every year they are no longer as rare as previously thought [1, 2]. Early diagnosis is crucial to limit PID-associated morbidity and mortality, as well as improve quality-of-life (QoL) [3].

Prior studies have highlighted that practitioners struggle to diagnose PID’s in clinical practice [4–6].

Physician education and public awareness campaigns have played a significant role in improving the diagnosis of these diseases [7]. In Eastern Europe, such programs have been implemented within the framework of the J Project under the leadership of prof. Lazlo Marodi [8–9]. At the beginning of 2004, there was a large difference in the PID diagnoses and patient care in Eastern and Western European countries, primarily due to low physician awareness and low access to genetic testing [9]. Implementation of clinical training and education (such as J Project meetings in different counties), availability of genetic testing for patients with suspected PID allowed improving the diagnosis and medical care of PID patients in Eastern Europe.

The aim of our study was to evaluate the knowledge about PIDs among physicians before and after the implementation of an educational program.

## Materials and method

A survey among physicians of different specialties to evaluate their knowledge about PIDs was conducted twice, in 2016 and in 2019, before and after the implementation of an educational program. The study involved physicians of Ternopil region, Ukraine.

To improve detection of PIDs in Ternopil region, we developed a project “Implementation of the Model of Combining Physician Education and Public Awareness with the Infrastructure to Diagnose Primary Immunodeficiency Diseases in Children in Western Ukraine”, which was supported by the Jeffrey Modell Foundation (JMF).

From February 2017 to February 2019, we organized lectures, workshops and trainings for primary care physicians (pediatricians and general practitioners / family physicians) on early PID detection in every district of Ternopil region and the city of Ternopil. In total, 15 districts were covered within the first year of the project. Overall, 540 physicians and nurses attended the lectures and workshops, including primary care physicians: pediatricians, general practitioners (GPs) and sub specialists. These meetings took place primarily in district hospitals, with the main topics of discussion involving PID clinical symptoms, warning signs, and main stages in laboratory diagnostics. Particular attention was paid to the most common PIDs of our region: Nijmegen breakage syndrome (NBS), Di George syndrome (DGS), and ataxia-telangiectasia (AT). Educational materials on PID warning signs and testing stages developed by JMF Advisory Board were translated and presented in Ukrainian. The members of our team also have created and produced a desk-top calendar with pages dedicated to highlighting warning signs of PIDs, diagnostic stages, normal levels of immunoglobulins and subpopulations of lymphocytes, as well as the main diagnostic criteria for the most common PIDs. We also have developed diagnostic algorithms and created the guidelines aimed at primary care physicians and specialists “Diagnostic algorithms for primary immunodeficiencies.” All these educational materials were printed and distributed among the physicians for free.

During the second year of our project, we focused our efforts on training medical sub-specialists on early PID symptoms and detection. We have analyzed the causes of delay in PID diagnosis in our region and defined the target audience. We have organized workshops for the sub specialists targeting specific disease symptoms that they could face in their practice. For example, the workshop “Cytopenia in newborns as a sign of PID” was presented to neonatologists, “Recurrent infections: when you should think about primary immunodeficiency” to pediatric infectious diseases specialists and pulmonologists, “Microcephaly in children: when you should think about Nijmegen breakage syndrome?” to neurologists, etc. In total, 270 sub specialists (pulmonologists, pediatric neurologists, otolaryngologists, gastroenterologists, pediatric endocrinologists, pediatric rheumatologists, allergists, neonatologists, dentists, pediatric hematologists, surgeons, pediatric infectious disease specialist, etc.) attended the lectures and presentations. We also presented the algorithms on PID diagnostics at conferences and meetings for sub specialists. These lectures were also opened to primary care physicians, and in total 550 of them attended the lectures during the second year of the project.

In total, in two years we organized 31 educational meetings, including primary care physicians, pediatricians, GPs (21) and sub specialists (10).

We compared responses from 82 doctors who participated in the 2016 survey: 42 pediatricians, 25 GPs and 15 physicians of pediatric sub-specialties (endocrinologists, infectious diseases specialists, pulmonologists, neurologists), with 67 doctors who took part in the 2019 survey: 34 pediatricians, 21 GPs and 12 physicians of pediatric sub-specialties. Each of the survey participants attended at least one educational event.

The questionnaire was developed by the immunologists Dr. Aleksandra Lewandowicz-Uszynska and her team (Wroclaw Medical University, Poland). It includes questions on basic knowledge about PIDs: warning signs developed by the JMF Medical Advisory Board, general signs of PIDs (recurrent infections, autoimmunity, allergy, malignancy), specific signs of the most common PIDs in Ternopil region (NBS, DGS, AT, CVID), principles of treatment and vaccination. The Ukrainian version of the questionnaire was fully cross-culturally adapted from the original Polish version with three forward and three backward translations. A final version was linguistically validated and approved by the copyright owner Dr. Aleksandra Lewandowicz-Uszynska. The questionnaire was validated using the scale internal consistency reliability by calculating Cronbach’s alpha coefficient (Cronbach’s alpha = 0.8773).

The survey included a questionnaire of 25 questions (Table 1). The questionnaire can be divided into four sections: warning signs in children (4 questions) and adults (2 questions); general signs of PIDs (5 questions); specific signs of PIDs (9 questions); treatment strategies and immunization of patients with PID (5 questions). Most questions were of ‘true’ or ‘false’ type. Two questions had two possible answers and two questions had three. In total, the physicians had to give 31 answers.

**Table 1:** Comparison of the number and percentage of correct answers, given by the surveyed physicians taking part in the study.

No.	Questions	2016 n = 82		2019 n = 67		P-value
		Correct answers				
		n	%	n	%	
1.	PIDs occur only in children	28	34.1	49	73.1^a^	<0.0001
2.	Telangiectasia may be specific to:					
	a) hepatic insufficiency,	50	61.0	56	83.6*	0.0024
	b) ataxia-telangiectasia syndrome (Louis-Bar syndrome)	44	53.7	55	82.1*	0.0003
3.	The absence of thymus confirms Di George syndrome	41	50.0	39	58.2	0.3175
4.	Common variable immunodeficiency (CVID) is most often diagnosed in children	15	18.3	16	23.9	0.4032
5.	Oncological diseases can be a sign of PID	50	61.0	53	79.1*	0.0172
6.	AFP (alpha-fetoprotein) appears in high concentrations in A-T syndrome	39	47.6	47	70.1*	0.0055
7.	Four or more new ear infections within 1 year may be a warning sign of PID	67	81.7	58	86.6	0.4221
8.	Failure of a child to gain weight normally may be a sign of PID	47	57.3	65	97.0*	<0.0001
9.	Repeated abscesses of skin and organs (without damage to the tissue integrity caused by trauma) may be a sign of PID	60	73.2	62	92.5*	0.0023
10.	Numerous (6 and more) of ‘coffee-with-milk’ colored spots are specific to:					
	a) Nijmegen breakage syndrome (NBS)	28	34.1	59	88.1*	<0.0001
	b) Louis-Bar syndrome	15	18.3	58	86.6*	<0.0001
	c) Bruton's agammaglobulinemia	52	63.4	53	79.1*	0.0368
11.	Two or more cases of pneumonia in a year may be the only clinical manifestation of PID	38	46.3	43	64.2*	0.0297
12.	Four or more episodes of infection (otitis, bronchitis, pneumonia) in an adult patient may be a sign of PID	56	68.3	56	83.6*	0.0316
13.	In adults, two or more cases of pneumonia (radiographically confirmed) within three years may be a sign of PID	58	70.7	45	67.2	0.6391
14.	Children diagnosed with microcephaly should undergo genetic testing	30	36.6	25	37.3	0.9260
15.	Infections with atypical localization or caused by atypical pathogens may be a sign of PID	67	81.7	58	86.6	0.4221
16.	Dysmorphic facial features are specific to:					
	а) common variable immunodeficiency (CVID)	64	78.0	55	82.1	0.5406
	b) DiGeorge syndrome	31	37.8	57	85.1*	<0.0001
	c) Nijmegen breakage syndrome	27	32.9	61	91.0*	<0.0001
17.	The only method of treatment for PID with antibody deficiency is therapy with intravenous or subcutaneous immunoglobulin agents	82	100	66	98.5	0.2670
18.	Normal levels of leukocytes (WBC), hemoglobin, platelets, HCT are sufficient to exclude neutropenia	66	80.5	52	77.6	0.6670
19.	Live vaccines are contraindicated for patients with NBS	59	72.0	57	85.1	0.0550
20.	Inflammation+ thrombocytopenia + eczema may be the signs of:					
	a) Wiskott-Aldrich syndrome	55	67.1	61	91.0*	0.0005
	b) atopic dermatitis	58	70.7	59	88.1*	0.0104
21.	In cases of Nijmegen syndrome chest X-ray examination is allowed	9	11.0	37	55.2*	<0.0001
22.	Live vaccines can be administered to children with severe PID	82	100	64	95.5	0.0529
23.	Vaccination against pneumococcus should be given to children with PID that have retained the ability to synthesize antibodies (within the risk group)	63	76.8	59	88.1	0,0767
24.	All adults with primary and secondary asplenia should be vaccinated against pneumococcus and meningococcus	36	43.9	57	85.1*	<0.0001
25.	Autoimmune diseases are much more common in patients with PID	64	78.0	58	86.6	0.1793
	Total	1481	58.3	1640	79.0*	<0.0001

* the difference is statistically significant

The questionnaires were distributed on-site during work hours. They were offered to the doctors who were in the workplace on the day of the survey. We used the paper version of the questionnaire and the initial contact with the potential participants was not made on the Internet. The survey was announced for the potential participants during the regular morning meetings either on the day of the survey, or the day before. We did not offer any incentive for the participants. The surveys distributed in 2016 and in 2019 were identical. Items were alternated. The survey was 1 page, 25 items. Only completed questionnaires were analyzed.

Verbal consent was obtained before conducting the questionnaire survey; the participants were informed why the information will be collected and how it will be used. Prior to handing in the questionnaire, a statement was read to the participants informing them that their participation was voluntary and that their answers were anonymous and confidential.

Ethical approval for the study was provided by the scientific ethics committee of I. Horbachevsky Ternopil State Medical University (protocol number 33). The study conformed to the principles outlined in the WMA Declaration of Helsinki.

The results were analyzed using standard procedures with Statistica StatSoft 6.0 software package. The distribution of variables was assessed by Chi-square test and the Fisher’s exact test. Significance level of the tests was set at P-value <0.05.

## Results

There were no significant differences in mean age of the respondents in 2016 and 2019. The age ranged from 25 to 69 years in 2016, and from 25 to 73 years old in 2019. The majority of the surveyed physicians were women (65 (79.2%) in 2016, and 55 (82.1%) in 2019). The female predominance matches physician demographics in Ukraine.

The comparison of the number and percentage of correct answers given by all respondents is presented in Table 1.

A significantly higher percentage of respondents in 2019 knew that PIDs did not occur only in children (73.1% versus 34.1% in 2016, P<0.0001). A significantly higher percentage of physicians in 2019 knew that failure to gain weight normally could be a sign of a PID (97.0% versus 57.3% in 2016, P<0.0001). In 2019 the doctors demonstrated better knowledge about specific features of Nijmegen breakage syndrome, DiGeorge syndrome and ataxia-telangiectasia syndrome. The majority of surveyed physicians in 2019 knew that numerous (6 and more) ‘coffee-with-milk’ colored spots are specific to NBS and to Louis-Bar syndrome (88.1% versus 34.1% in 2016, P<0.001, and 86.6% versus 18.3% in 2016, P<0.0001, respectively). Following the educational program, the majority of surveyed respondents also knew that dysmorphic facial features are specific to DiGeorge syndrome and Nijmegen breakage syndrome (85.1% versus 37.8% in 2016, P<0.0001, and 91.0% versus 32.9% in 2016, P<0.0001, respectively). A much larger proportion of the surveyed physicians became aware that chest X-ray examination is not allowed for Nijmegen syndrome (55.2% versus 11.0% in 2016, P<0.0001). Better knowledge was demonstrated about vaccination against pneumococcus and meningococcus in adults with primary and secondary asplenia (85.1% versus 43.9% in 2016, P<0.0001).

Overall, the percentage of correct answers to all survey questions in 2019 has increased compared to 2016 (79.0% versus 58.3% in 2016, P<0.0001).

The number of correct answers given by the physicians corresponding to their specialties is presented in Table 2.

**Table 2:** Comparison of the number and percentage of correct answers given by the surveyed physicians corresponding to their specialty.

No.	Physicians specialty	n of physicians		n of answers		Correct answers				P-value
						n	%	n	%	
		2016	2019	2016	2019	2016		2019		
1.	Pediatricians	42	34	1302	1054	785	60.3	851	80.7	<0.0001
2.	General practitioners	25	21	775	651	423	54.6	495	76.0	<0.0001
3.	Pediatric sub-specialists	15	12	465	372	273	58.7	294	79.0	<0.0001

An increase in the percentage of correct answers was noted among the surveyed doctors of all specialties. Pediatricians gave more than 80% of correct answers, other groups also showed progress, although no significant difference was found between pediatricians and other specialty physicians.

Analysis of the answers was conducted in the context of question sections. The number of correct answers to the questions about the warning signs of PIDs in children and adults is presented in Table 3.

**Table 3:** Comparison of the number and percentage of correct answers for the questions about warning signs in children and adults.

No.	Question	2016		2019	
		n = 82		n = 67	
		n	%	n	%
	**Warning signs in children**	212	64.6	228	85.1*
1.	Four or more new ear infections within 1 year may be a warning sign of PID	67	81.7	58	86.6
2.	Failure of a child to gain weight normally may be a sign of PID	47	57.3	65	97.0
3.	Two or more cases of pneumonia in a year may be the only clinical manifestation of PID	38	46.3	43	64.2
4.	Repeated abscesses of skin and organs (without damage to the tissue integrity caused by trauma) may be a sign of PID	60	73.2	62	92.5
	**Warning signs in adults**	114	69.5	101	75.4
1.	Four or more episodes of infection (otitis, bronchitis, pneumonia) in an adult patient may be a sign of PID	56	68.3	56	83.6
2.	In adults, two or more cases of pneumonia (radiographically confirmed) within three years may be a sign of PID	58	70.7	45	67.2

*P<0.0001

There was a significant increase in the number of correct answers about the warning signs of PIDs in children (85.1% versus 64.6% in 2016, P<0.0001). An increase in correct answers was noted for all questions of this group. There was no significant difference in improving the correct answers to warning signs in adults.

The comparison of the answers to the questions about general signs of PIDs is presented in Table 4. The results demonstrate a big improvement in the knowledge of some aspects (for example, whether PIDs occur only in children), although there were no significant differences between the percentage of correct answers to all three questions of this block in 2016 and 2019 years.

**Table 4:** Comparison of the number and percentage of correct answers to the questions about general signs of PIDs.

No	Question	2016		2019	
		n = 82		n = 67	
		n	%	n	%
1.	PIDs occur only in children	28	34.1	49	73.1
2.	Common variable immunodeficiency (CVID) is most often diagnosed in children	15	18.3	16	23.9
3.	Oncological diseases can by a sign of PID	50	61.0	53	79.1
4.	Autoimmune diseases are much more common in patients with PID	64	78.0	58	86.6
5.	Infections with atypical localization or caused by atypical pathogens may be a sign of PID	67	81.7	58	86.6
	Total	224	54.6	234	69.9*

*P<0.0001

The number and percentage of correct answers to the questions about specific signs of certain PIDs is presented in Table 5.

**Table 5:** Comparison of the number and percentage of correct answers for the questions about specific signs of PIDs.

N	Question	2016		2019	
		n = 82		n = 67	
		n	%	n	%
1.	Telangiectasia may be specific to:				
	a) hepatic insufficiency,	50	61.0	56	83.6
	b) ataxia-telangiectasia syndrome (Louis-Bar syndrome)	44	53.7	55	82.1
2.	The absence of thymus confirms Di George syndrome	41	50.0	39	58.2
3.	Numerous (6 and more) of ‘coffee-with-milk’ colored spots are specific to:				
	a) Nijmegen breakage syndrome (NBS)	28	34.1	59	88.1
	b) Louis-Bar syndrome	15	18.3	58	86.6
	c) Bruton's agammaglobulinemia	52	63.4	53	79.1
4.	Dysmorphic facial features are specific to:				
	a) common variable immunodeficiency (CVID)	64	78.0	55	82.1
	b) DiGeorge syndrome	31	37.8	57	85.1
	c) Nijmegen breakage syndrome	27	32.9	61	91.0
**5.**	Inflammation+ thrombocytopenia + eczema may be the signs of:				
	a) Wiskott-Aldrich syndrome	55	67.1	61	91.0
	b) atopic dermatitis	58	70.7	59	88.1
6.	In cases of Nijmegen syndrome chest X-ray examination is allowed	9	11.0	37	55.2
7.	Normal levels of leukocytes (WBC), hemoglobin, platelets, HCT are sufficient to exclude neutropenia	66	80.5	52	77.6
8.	AFP (alpha-fetoprotein) appears in high concentrations in A-T syndrome	39	47.6	47	70.1
9.	Children diagnosed with microcephaly should undergo genetic testing	30	36.6	25	37.3
	Total:	609	49.5	774	77.0*

*P<0.0001

In 2016,this block of questions received the lowest percentage of correct answers, less than 50%, while in 2019, there was a significant increase in the number of correct answers (77.0% versus 49.5% in 2016, P <0.0001). Most of the questions in this block received an increased number of correct answers.

The analysis of the answers to the questions about treatment and vaccination of children with PID in 2016 and in 2019 years is presented in Table 6.

**Table 6:** Comparison of the number and percentage of correct answers for the questions about treatment and vaccination of children with PID.

N	Question	2016		2019	
		n = 82		n = 67	
		n	%	n	%
1.	The only method of treatment for PID with antibody deficiency is therapy with intravenous or subcutaneous immunoglobulin agents	82	100	66	98.5
2.	Live vaccines are contraindicated for patients with NBS	59	72.0	57	85.1
3.	Live vaccines can be administered to children with severe PID	82	100	64	95.5
4.	Vaccination against pneumococcus should be given to children with PID that have retained the ability to synthesize antibodies (within the risk group)	63	76.8	59	88.1
5.	All adults with primary and secondary asplenia should be vaccinated against pneumococcus and meningococcus	36	43.9	57	85.1
	Total:	322	78.5	303	90.4*

*P<0.0001

In 2019 questions of this group received more than 85% of correct answers, and the overall this block of questions had the most correct answers (90.4%).

## Discussion

Improving knowledge of the physicians is one of the ways to improve PID diagnosis. Our study showed that after implementation of the educational program, correct answers to all survey questions have increased by more than 20% (79.0% versus 58.3%, P<0.0001), which can indicate the effectiveness of these activities. No significant difference was found between pediatricians and other specialty physicians (GPs and pediatric sub specialists). This is in contrast to a study in Iran: a survey involved 50 general practitioners, 182 pediatric specialists, and 49 pediatric sub specialists demonstrated lack of awareness on PIDs in physicians (the mean score 55.9%) and difference between physicians groups: pediatric sub specialists significantly over performed other participants [10]. On the other hand, a Brazilian study demonstrated that pediatricians had a better knowledge about PIDs than clinicians and surgeons [11].

In a previous study, we have shown that there was a low knowledge about the specific signs of PIDs, in particular verification of Nijmegen breakage syndrome, ataxia-telangiectasia and Di George syndrome [4]. Therefore, we aimed to improve the knowledge of warning and specific signs of PIDs. Particular attention was paid to the diagnosis of Nijmegen breakage syndrome and Di George syndrome, which are the most common in Ternopil region [12–14]. In this study, we see a significant increase in the percentage of correct answers (by 27.5%) in this block of questions. However, for some questions, the percentage of correct answers remains low (less than 50%). In particular, the majority doctors think that CVID is predominantly diagnosed in children. Although the percentage of correct answers to the questions addressing chest X-ray examination in Nijmegen syndrome cases has significantly increased (P <0.0001), it still remains low (55.2%). There was a significant increase in the number of correct answers about the warning signs of PIDs in children (85.1% versus 64.6% in 2016, P<0.0001), however there was no significant improvement in the knowledge about warning signs in adults. This is consistent with a study from Brazil, which also revealed low knowledge of warning signs for primary immunodeficiency [11].

The effectiveness of an educational program to improve PID diagnosis has been demonstrated in other studies [7–9]. The main goal of J Project, which brings together countries in Eastern and Central Europe, is to improve PID diagnosis, and one of the main strategies for achieving this goal is education campaign. Ukraine is one of the most active participants of the J Project [9], with educational conferences within the framework of the project held annually in different regions of Ukraine. Our particular project had a more regional focus; we wanted to bring knowledge about PIDs to every primary care physician and every remote physician’s office.

The impact of physician education and public awareness campaign from the Jeffrey Modell Foundation on early diagnosis and management of PID was previously assessed, finding that the number of diagnostic tests performed by participating physicians at Jeffrey Modell Centers increased annually by nearly 5 times over a 4 year period [7].

The effectiveness of implementing an educational program was demonstrated in a study from Mexico [15], which found improved diagnostic outcomes at the regional level. They estimated that 75 physicians need to be taught to get one PID patient referral [15]. Therefore, education is a long and painstaking process, but it is very necessary to improve the diagnosis of PIDs.

Our previous study has shown that implementation of an educational program resulted in the increase of the number of detected PIDs immediately afterwards almost threefold, and compared to the two years prior to the awareness campaign (2015–2016) almost fivefold (2 cases per year before versus 9.5 cases per year during the awareness campaign) [14]. The number of children with suspected PIDs referred by pediatric sub specialists has almost doubled in the second year of the program (41 vs. 22) [14]. This result is consistent with those of previous studies measuring the impact of physician education and public awareness campaigns in Jeffrey Modell Centers Network of 39 countries and 120 cities worldwide [7]. The highest percentage of diagnosed PIDs was also in children referred by specialist doctors (73.7%) [14].

Among the children that were referred with suspected PID 12.7% of the cases were confirmed [14], which is consistent with the results of other study which showed a PID prevalence 9.9% among those referred for evaluation [15].

The mean delay from initial symptoms to diagnosis in the patients from our center was 31.5 months (2.6 years), ranging from one to 156 months [16]. Other studies reported the mean delay from initial symptoms to a referral for medical advice at 3.7 years [15]; the mean, depending on a PID category, was from 6 to 56 months, and the range from 1 to 134 months [17]. In our previous study, the longest delay to a diagnosis was in a newly diagnosed 16 year-old patient with AT [16]. The ESID registry data also points out to delays in the diagnosis of AT, its mean (4.38 years) being one of the largest [18]. On the other hand, children with NBS and DGS received timely diagnoses within the first months after birth [16].

The importance of developing and implementing training programs for physicians, nurses and students to improve the diagnosis and treatment of diseases, the importance of teamwork in ensuring the success of diagnostic and therapeutic manipulations is highlighted in other papers [19–20].

## Conclusion

The survey we conducted has shown significant improvement of physicians’ awareness concerning PIDs after implementation of an educational program.

Particular progress has been made in improving medical knowledge about warning signs and specific signs of PIDs. Enhancing of doctors’ knowledge on PIDs will improve early detection of PIDs and their medical care.

## Supporting information

S1 File Primary immunodeficiency survey. (DOCX)

S2 File Ankieta dotycząca pierwotnych niedoborów odporności. (DOC)

S3 File Анкета «Первинні імунодефіцити». (DOCX)

S1 Data (XLSX)

S2 Data (XLSX)
