# Supplementary material for: Evaluation of awareness about primary immunodeficiencies among physicians before and after implementation of the educational program: A longitudinal study
Source: PLoS One. 2020 May 29;15(5):e0233342. doi: 10.1371/journal.pone.0233342 (PMC7259605; doi:10.1371/journal.pone.0233342)
Supplement: S1 File — (DOCX) [file pone.0233342.s001.docx]

Primary Immunodeficiency Survey

| Speciality | age | sex |
| --- | --- | --- |
|  |  |  |

Please tick the answers X

|  | Question | Yes | No |
| --- | --- | --- | --- |
| 1. | PIDs occur only in children |  |  |
| 2. | Telangiectasia may be specific to:  а) hepatic insufficiency,  b) ataxia-telangiectasia syndrome (Louis-Bar syndrome) |  |  |
| 3. | The absence of thymus confirms Di George syndrome |  |  |
| 4. | Common variable immunodeficiency (CVID) is most often diagnosed in children |  |  |
| 5. | Oncological diseases can be a sign of PID |  |  |
| 6. | AFP (alpha-fetoprotein) appears in high concentrations in A-T syndrome |  |  |
| 7. | Four or more new ear infections within 1 year may be a warning sign of PID |  |  |
| 8. | Failure of a child to gain weight normally may be a sign of PID |  |  |
| 9. | Repeated abscesses of skin and organs (without damage to the tissue integrity caused by trauma) may be a sign of PID |  |  |
| 10. | Numerous (6 and more) of ‘coffee-with-milk’ colored spots are specific to:  а) Nijmegen breakage syndrome (NBS)  b) Louis-Bar syndrome  c) Bruton's agammaglobulinemia |  |  |
| 11. | Two or more cases of pneumonia in a year may be the only clinical manifestation of PID |  |  |
| 12. | Four or more episodes of infection (otitis, bronchitis, pneumonia) in an adult patient may be a sign of PID |  |  |
| 13. | In adults, two or more cases of pneumonia (radiographically confirmed) within three years may be a sign of PID |  |  |
| 14. | Children diagnosed with microcephaly should undergo genetic testing |  |  |
| 15. | Infections with atypical localization or caused by atypical pathogens may be a sign of PID |  |  |
| 16. | Dysmorphic facial features are specific to:  а) common variable immunodeficiency (CVID)  b) DiGeorge syndrome  c) Nijmegen breakage syndrome |  |  |
| 17. | The only method of treatment for PID with antibody deficiency is therapy with intravenous or subcutaneous immunoglobulin agents |  |  |
| 18. | Normal levels of leukocytes (WBC), hemoglobin, platelets, HCT are sufficient to exclude neutropenia |  |  |
| 19. | Live vaccines are contraindicated for patients with NBS |  |  |
| 20. | Inflammation+ thrombocytopenia + eczema may be the signs of:  а) Wiskott-Aldrich syndrome  b) atopic dermatitis |  |  |
| 21. | In cases of Nijmegen syndrome chest X-ray examination is allowed |  |  |
| 22. | Live vaccines can be administered to children with severe PID |  |  |
| 23. | Vaccination against pneumococcus should be given to children with PID that have retained the ability to synthesize antibodies (within the risk group) |  |  |
| 24. | All adults with primary and secondary asplenia should be vaccinated against pneumococcus and meningococcus |  |  |
| 25. | Autoimmune diseases are much more common in patients with PID |  |  |
