# Supplementary material for: Evaluation of awareness about primary immunodeficiencies among physicians before and after implementation of the educational program: A longitudinal study
Source: PLoS One. 2020 May 29;15(5):e0233342. doi: 10.1371/journal.pone.0233342 (PMC7259605; doi:10.1371/journal.pone.0233342)
Supplement: S2 File — (DOC) [file pone.0233342.s002.doc]

Ankieta dotycząca Pierwotnych Niedoborów Odporności

| specjalizacja | wiek | płeć |
| --- | --- | --- |
|  |  |  |

Odpowiedzi proszę zaznaczyć X

|  | Pytanie | tak | nie |
| --- | --- | --- | --- |
| 1 | Pierwotne niedobory odporności występują tylko u dzieci |  |  |
| 2 | Teleangiektazje mogą być charakterystyczne dla:   - niewydolności wątroby, - Zespołu ataksja-teleangiektazja (A-T) |  |  |
| 3 | W Zespole DiGeorga nie stwierdza się obecności grasicy |  |  |
| 4 | Pospolity zmienny niedobór odporności rozpoznaje się najczęściej u dzieci |  |  |
| 5 | Choroba nowotworowa może być objawem PNO |  |  |
| 6 | AFP (alfa-fetoproteina) występuje w podwyższonych stężeniach w Zespole A-T |  |  |
| 7 | Cztery lub więcej zapaleń uszu może być objawem alarmowym PNO |  |  |
| 8 | Zahamowanie przyrostu masy ciała może być objawem PNO |  |  |
| 9 | Powtarzające się ropnie skórne i narządowe (bez przerwania ciągłości tkanek wskutek urazu) mogą być objawem PNO |  |  |
| 10 | Plamy „caffe-ai-lait” występujące w liczbie 6 i powyżej występują szczególnie często w:   - Zespole Nijmegen - Zespole A-T - Agammaglobulinemii typu Brutona |  |  |
| 11 | Dwa lub więcej zapalenia płuc w ciągu roku może być jedynym klinicznym objawem PNO |  |  |
| 12 | Cztery lub więcej zakażenia (uszu, oskrzeli, płuc) u osoby dorosłej może być objawem PNO |  |  |
| 13 | Dwa lub więcej zapalenia płuc (potwierdzone w RTG) w ciągu 3 lat u osoby dorosłej może być objawem PNO |  |  |
| 14 | Tylko wybrane dzieci, u których stwierdza się małogłowie należy skierować na badania genetyczne |  |  |
| 15 | Zakażenia o niezwykłej lokalizacji lub wywołane przez niezwykłe patogeny mogą być objawem sugerującym PNO |  |  |
| 16 | Dysmorfię twarzy stwierdza się w :   - Pospolitym zmiennym niedoborze odporności (CVID) - Zespole DiGeorga - Zespole Nijmegen |  |  |
| 17 | Jedną z możliwości leczenia PNO (przebiegających z deficytem przeciwciał) jest terapia dożylnymi lub podskórnymi preparatami immunoglobulin |  |  |
| 18 | Prawidłowy wynik morfologii WBC, Hgb, PTL, HCT jest wystarczający do wykluczenia neutropenii |  |  |
| 19 | W zespołach NBS nie należy szczepić żywymi szczepionkami |  |  |
| 20 | Zakażenia +Trombocytopenia+ egzema może występować w:   - Zespole Wiskott-Aldrich - Atopowym zapaleniu skóry |  |  |
| 21 | W zespołach NBS wolno wykonywać badania RTG |  |  |
| 22 | Dzieci z głębokimi PNO wolno szczepić żywymi szczepionkami |  |  |
| 23 | Szczepienia przeciwko pneumokokom należy wykonać u dzieci z PNO które zachowały zdolność syntezy przeciwciał w ramach grup ryzyka |  |  |
| 24 | Wszystkich dorosłych z pierwotną lub wtórną asplenią należy szczepić przeciwko: pneumokokom, meningokokom, |  |  |
| 25 | Choroby autoimmunizacyjne zdecydowanie częściej występują u osób z PNO |  |  |
