# Supplementary material for: Evaluation of awareness about primary immunodeficiencies among physicians before and after implementation of the educational program: A longitudinal study
Source: PLoS One. 2020 May 29;15(5):e0233342. doi: 10.1371/journal.pone.0233342 (PMC7259605; doi:10.1371/journal.pone.0233342)
Supplement: S3 File — (DOCX) [file pone.0233342.s003.docx]

**Анкета «Первинні імунодефіцити»**

| Спеціальність | вік | стать |
| --- | --- | --- |
|  |  |  |

Будь ласка, відмітьте вашу відповідь X

| п/п | Питання | Відповідь | |
| --- | --- | --- | --- |
|  |  | так | ні |
| 1. | Первинні імунодефіцити зустрічаються тільки у дітей |  |  |
| 2. | Телеангектазії можуть бути характерними для:  а) печінкової недостатністі,  б) захворювання атаксії-телеангіектазії (синдрому Луї-Бар) |  |  |
| 3. | Відсутність тимусу підтверджує синдром Ді-Джорджа |  |  |
| 4. | Загальний варіабельний імунодефіцит (ЗВІД) найчастіше діагностується у дітей |  |  |
| 5. | Онкозахворювання можуть бути ознакою ПІД |  |  |
| 6. | Альфа-фетопротеїнемія (AFP) виступає в підвищених концентраціях при атаксії-телеангіектазії (А-Т) |  |  |
| 7. | Чотири або більше отитів впродовж року можуть бути ознакою, що насторожує щодо ПІД |  |  |
| 8. | Затримка прибавки маси тіла може бути ознакою ПІД |  |  |
| 9. | Повторні абсцеси шкіри та органів (без переривання цілісності тканин через травму) можуть бути ознакою ПІД |  |  |
| 10. | Плями "кава-з-молоком", які виступають в кількості 6 і більше, особливо поширені при:  а) захворюванні Ніймегена (NBS)  б) захворюванні Луї-Бар  в) агамглобулінемії типу Брутона |  |  |
| 11. | Два або більше запалень легень впродовж року можуть бути єдиним клінічним проявом ПІД |  |  |
| 12. | Чотири або більше запалень (отити, бронхіти, пневмонії) в дорослих осіб можуть бути ознакою ПІД |  |  |
| 13. | Дві і більше пневмонії, (підтверджені рентгенологічно) впродовж трьох років в дорослих можуть бути ознакою ПІД |  |  |
| 14. | Дітей, в яких підтверджено мікроцефалію потрібно направляти на генетичне обстеження |  |  |
| 15. | Інфекції з нетиповою локалізацією або спричинені нетиповим патогеном можуть бути ознакою ПІД |  |  |
| 16. | Дисморфія обличчя характерна для:  а) загального варіабельного імунодефіциту (ЗВІД)  б) синдрому Ді-Джорджа  в) синдрому Ніймегена |  |  |
| 17. | Одним із методів лікування ПІД, які перебігають з дефіцитом антитіл, є терапія внутрішньовенного або підшкірного введення препаратів імуноглобуліну |  |  |
| 18. | Нормальні показники морфології лейкоцитів (WBC), гемоглобіну (Hb), тромбоцитів (PTL), гематокриту (HCT) є достатнім для виключення нейтропенії |  |  |
| 19. | При синдромі Ніймегена протипоказане введення живих вакцин |  |  |
| 20. | Запалення+тромбоцитопенія+екзема можуть виступати проявами:  а) синдрому Wiskott-Aldrich  б) атопічного запелення шкіри |  |  |
| 21. | При синдромі Ніймегена можна проводити рентгенографію органів грудної порожнини |  |  |
| 22. | Дітям з тяжкими ПІД можна вводити живі вакцини |  |  |
| 23. | Вакцинацію проти пневмококів належить виконувати дітям з ПІД, які зберегли здатність до синтезу антитіл, в рамках групи ризику |  |  |
| 24. | Всіх дорослих з первинною або вторинною аспленією потрібно щепити проти: пневмококів, менінгококів |  |  |
| 25. | Аутоімунні хвороби набагато частіше виявляються в осіб з ПІД |  |  |
